# Guidelines for experimental models of myocardial ischemia and infarction

**DOI:** 10.1152/ajpheart.00335.2017

**Published:** 2018-01-12

**Authors:** Merry L. Lindsey, Roberto Bolli, John M. Canty, Xiao-Jun Du, Nikolaos G. Frangogiannis, Stefan Frantz, Robert G. Gourdie, Jeffrey W. Holmes, Steven P. Jones, Robert A. Kloner, David J. Lefer, Ronglih Liao, Elizabeth Murphy, Peipei Ping, Karin Przyklenk, Fabio A. Recchia, Lisa Schwartz Longacre, Crystal M. Ripplinger, Jennifer E. Van Eyk, Gerd Heusch

**Affiliations:** ^1^Mississippi Center for Heart Research, Department of Physiology and Biophysics, University of Mississippi Medical Center, Jackson, Mississippi; ^2^Research Service, G. V. (Sonny) Montgomery Veterans Affairs Medical Center, Jackson, Mississippi; ^3^Division of Cardiovascular Medicine and Institute of Molecular Cardiology, University of Louisville, Louisville, Kentucky; ^4^Division of Cardiovascular Medicine, Departments of Biomedical Engineering and Physiology and Biophysics, The Veterans Affairs Western New York Health Care System and Clinical and Translational Science Institute, Jacobs School of Medicine and Biomedical Sciences, University at Buffalo, Buffalo, New York; ^5^Baker Heart and Diabetes Institute, Melbourne, Victoria, Australia; ^6^The Wilf Family Cardiovascular Research Institute, Department of Medicine (Cardiology), Albert Einstein College of Medicine, Bronx, New York; ^7^Department of Internal Medicine I, University Hospital, Würzburg, Germany; ^8^Center for Heart and Regenerative Medicine Research, Virginia Tech Carilion Research Institute, Roanoke, Virginia; ^9^Department of Biomedical Engineering, University of Virginia Health System, Charlottesville, Virginia; ^10^Department of Medicine, Institute of Molecular Cardiology, Diabetes and Obesity Center, University of Louisville, Louisville, Kentucky; ^11^HMRI Cardiovascular Research Institute, Huntington Medical Research Institutes, Pasadena, California; ^12^Division of Cardiovascular Medicine, Keck School of Medicine, University of Southern California, Los Angeles, California; ^13^Cardiovascular Center of Excellence, Louisiana State University Health Science Center, New Orleans, Louisiana; ^14^Harvard Medical School, Boston, Massachusetts; ^15^Division of Genetics and Division of Cardiovascular Medicine, Department of Medicine, Brigham and Women's Hospital, Boston, Massachusetts; ^16^Systems Biology Center, National Heart, Lung, and Blood Institute, National Institutes of Health, Bethesda, Maryland; ^17^National Institutes of Health BD2KBig Data to Knowledge (BD2K) Center of Excellence and Department of Physiology, Medicine and Bioinformatics, University of California, Los Angeles, California; ^18^Cardiovascular Research Institute and Departments of Physiology and Emergency Medicine, Wayne State University School of Medicine, Detroit, Michigan; ^19^Institute of Life Sciences, Scuola Superiore Sant'Anna, Fondazione G. Monasterio, Pisa, Italy; ^20^Cardiovascular Research Center, Lewis Katz School of Medicine, Temple University, Philadelphia, Pennsylvania; ^21^Heart Failure and Arrhythmias Branch, Division of Cardiovascular Sciences, National Heart, Lung, and Blood Institute, National Institutes of Health, Bethesda, Maryland; ^22^Department of Pharmacology, School of Medicine, University of California, Davis, California; ^23^The Smidt Heart Institute, Department of Medicine, Cedars Sinai Medical Center, Los Angeles, California; ^24^Institute for Pathophysiology, West German Heart and Vascular Center, University of Essen Medical School, Essen, Germany

**Keywords:** animal models, cardiac remodeling, heart failure, myocardial infarction, reperfusion, rigor and reproducibility

## Abstract

Myocardial infarction is a prevalent major cardiovascular event that arises from myocardial ischemia with or without reperfusion, and basic and translational research is needed to better understand its underlying mechanisms and consequences for cardiac structure and function. Ischemia underlies a broad range of clinical scenarios ranging from angina to hibernation to permanent occlusion, and while reperfusion is mandatory for salvage from ischemic injury, reperfusion also inflicts injury on its own. In this consensus statement, we present recommendations for animal models of myocardial ischemia and infarction. With increasing awareness of the need for rigor and reproducibility in designing and performing scientific research to ensure validation of results, the goal of this review is to provide best practice information regarding myocardial ischemia-reperfusion and infarction models.

Listen to this article’s corresponding podcast at ajpheart.podbean.com/e/guidelines-for-experimental-models-of-myocardial-ischemia-and-infarction/.

## INTRODUCTION

**Fig. 1.**
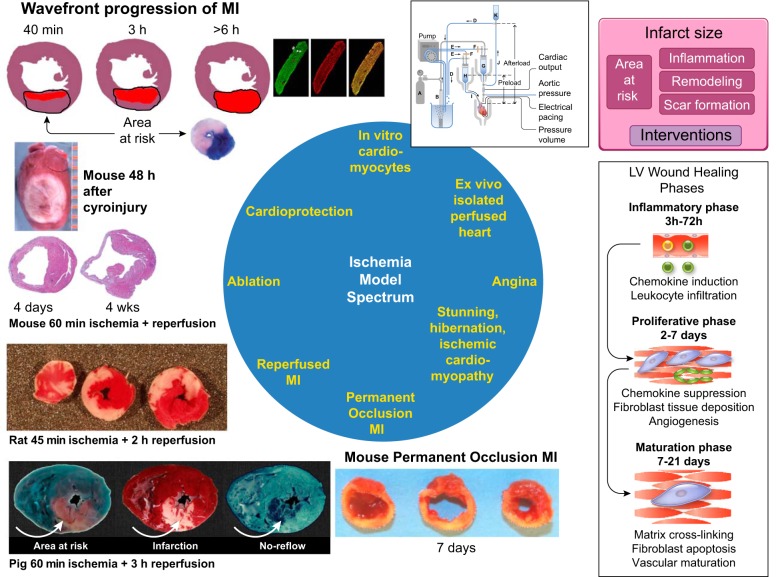
The spectrum of ischemia encapsulates in vitro, ex vivo, and in vivo models of ischemia that range from transient to prolonged in duration with acute to chronic consequences. The pig section (*bottom left*) is modified from Heusch (126). MI, myocardial infarction; LV, left ventricular;

Ischemia occurs when blood flow to the myocardium is reduced (129). Ischemia of prolonged duration induces myocardial infarction (MI), and MI is a common cause of heart failure (295). Ischemic cardiomyopathy is the most common cause of heart failure and can arise from remodeling after an acute ST segment elevation myocardial infarction (STEMI) from multiple small nontransmural infarctions or from chronic repetitive ischemia in the absence of infarction (15). Ischemia can range in its extent from low flow to total coronary occlusion, can be of short to long duration, can be successfully reversed by reperfusion in a timely manner or not reperfused at all, and can induce injury or provide cardioprotection. Likewise, there is a diverse variety of animal models to address each type of ischemia within this spectrum. Figure 1 shows the range of models that reflect the scale of ischemia and variety of models available to better understand how the heart responds to ischemia and the mechanisms whereby the heart can either adapt to ischemia or progress to failure.

Experimental models of myocardial ischemia serve two nearly opposing aims, both worthy of investigation. The first aim is to provide better mechanistic insight that cannot be obtained from a clinical situation. To achieve this aim, experimental studies may be reductionist with low direct applicability to the clinical situation (e.g., when using temporally induced cell specific over- or underexpression of a gene). The second aim is to provide mechanistic insight from an experimental study for translation to the clinical situation, and for this aim experimental models must replicate the clinical setting as closely as possible (127).

For cardiovascular science to continue advancing, experimental results should be reproducible and replicable, and rigorous experimental design is a fundamental element of reproducibility. Reproducibility refers to results that can be repeated by multiple scientists and is a means of validation across laboratories. Rigor refers to robust and unbiased experimental design, methodology, analysis, interpretation, and reporting of results. With increasing awareness by journals and granting agencies of the need for reproducibility and rigor in designing and performing scientific research in preclinical studies, the goal of this consensus article is to provide best practice information regarding myocardial ischemia and infarction models. The strengths and limitations of the different models are discussed, with a summary shown in Table 1. We also address ways to incorporate Animals in Research: Reporting In Vivo Experiments (ARRIVE) guidelines and similar standard operating procedures (168). The extensive reference list provided also serves as a resource for researchers new to the field.

**Table 1.** Comparison of different approaches with strengths and limitations for each method

Approach	End-Point Measurements	Strengths	Limitations and Pitfalls
In vitro cardiomyocytes	Cell viability (live-dead assay)	High throughput	Reductionist
	Type of cell death (i.e., apoptosis)	Isolate effects of hypoxia/reoxygenation on cardiomyocytes without other cell types or circulating factors	Cardiomyocyte viability may not predict changes in infarct size in vivo
			Adult cardiomyocyte culture technically challenging
Isolated perfused hearts	Infarct size per area at risk	Relatively easy and reproducible	Tissue edema
	Left ventricular function	Can study ischemia and reperfusion	May not fully represent the in vivo response
	Assessment of cardiac troponin I as a secondary cardiac injury index	Accurate measure of infarct size	Glucose as the sole substrate
		Ample sample for biochemistry	Limited stability
		Compatible with NMR studies	Excessive coronary flow
		Capacity for high throughput	Reductionist
		Neurohormonal factor independent	
		Eliminates confounding effect of intervention on systemic blood vessels or circulating factors	
Angina	Regional flow and function	Close to clinical situation	Technically complex; time and cost intensive
	Metabolism, morphology, molecular biology, nerve activity, rhythm		
Hibernation/stunning	Regional flow and function	Close to clinical situation	Technically complex; time and cost intensive
	Metabolism, morphology, molecular biology, rhythm		
Permanent occlusion MI	Inflammation, wound healing, scar formation, remote region myocytes, electrical activity	In the era of percutaneous coronary intervention, ~15–25% patients are not successfully reperfused in a timely manner (53, 104)	Does not reflect the reperfused MI patient response
		Robust remodeling response; large effect size	
Ischemia-reperfusion MI	Inflammation, wound healing, scar formation, myocyte viability, electrical activity	Close to clinical scenario	More technically challenging surgery
			Reperfusion injury can expand area of damage
Ablation	Inflammation, wound healing, scar formation, myocyte electrical activity	Geometrically defined lesion	Nonischemic lethal injury
		Infarct size/location independent of coronary anatomy	Mechanisms of cell death different from ischemia
Cardioprotection	Infarct size per area at riskLeft ventricular geometry and function; no reflow; circulating biomarkers such as cardiac troponin I	Mouse, rat, rabbit, and pig: models of low collateral flow (measurement of regional myocardial blood flow not required)Rat and rabbit: reliable infarct production; relatively high survival ratePig: mimics humans with low collateral flowDog: large amount of historical data; shows effect of intervention in the setting of variable collateral flow; mimics humans with high collateral flow	Mouse: small size; substantial variability requiring large *n* valuesRat, rabbit, pig, and dog: not high throughput; higher costRabbit, pig, and dog: potential for lethal arrhythmiasDog: variability in collateral perfusion (regional myocardial blood flow must be measured)

MI, myocardial infarction.

## IN VITRO AND EX VIVO MODELS

### Myocyte Cell Culture

#### Model rationale.

Isolated fresh or cultured cardiomyocytes can be used as a powerful in vitro model of ischemia-reperfusion (I/R), whereby ischemia is simulated with hypoxia and reperfusion with reoxygenation (H/R). This system allows precise control of the cellular and extracellular environment, notably the specific impact of hypoxia and reoxygenation on cardiomyocytes without confounding influences of other cell types (e.g., fibroblasts, endothelial cells, inflammatory/immune cells, and platelets) or circulating factors (e.g., hormones, neurotransmitters, and cytokines).

#### Variables measured.

The most common use of this model system is for in vitro testing of specific factors proposed to be involved in I/R injury or cardioprotection (31, 166, 227, 240, 244, 330). After H/R, cultured cardiomyocytes undergo apoptosis, accompanied by cytochrome *c* release and caspase activation or necrosis (200, 296). Thus, assays of cell viability are often performed to assess the role of a particular genetic or pharmacological intervention in exacerbating or protecting the cell from H/R-induced cell death. Viability may be measured with a variety of assays, including lactate dehydrogenase (LDH) release or propidium iodide exclusion as an indicator of membrane integrity (24, 31, 58). Apoptosis is assessed with TUNEL or annexin V staining (24, 58, 166). Mitochondrial damage, including disruption of mitochondrial membrane potential, is also a key component of cellular injury after H/R and may be assessed using fluorescent dyes, such as tetramethylrhodamine methyl ester (TMRM). The loss of mitochondrial membrane potential causes TMRM to leak from the mitochondria, decreasing fluorescence (24, 31). In addition, reactive oxygen species (ROS) have been implicated in H/R injury (275); thus, ROS production is another common assessment (24, 58, 124, 125, 227).

More detailed analyses of cardiomyocyte responses to H/R include assessments of morphology, contractile function (i.e., cell shortening), intracellular Ca^2+^ handling, and action potentials (124, 166, 207). Contractile function is an important but often overlooked variable in H/R assays, since contraction requires ~70% of total energy utilization within a myocyte (182). Many H/R studies use quiescent myocytes; however, markedly impaired recovery of myocyte function and increased cell death result when cells are stimulated to contract throughout the H/R protocol (207). For all variables assessed, technical replicates on the same sample should be performed to establish the variability of the measurement technique. Biological replicates often include measurements on plates or myocytes from the same heart/harvest. If these are to be treated as independent samples, *n* values for both the plate/myocyte number as well as heart/harvest number should be fully reported.

There is currently no standardized protocol for H/R in cultured cardiomyocytes, but cardiomyocyte source and H/R conditions must be carefully considered. Theoretically, freshly isolated adult cardiomyocytes are the ideal gold standard for H/R experiments (166, 207, 244), although neonatal cardiomyocytes (58, 227, 296), cardiac progenitor cells (12), induced pluripotent stem cell-derived cardiomyocytes (iPSC-CMs) (24, 31), and various cell lines such as H9c2 and HL-1 have been used (330). Of these, neonatal cardiomyocytes are most commonly used, due to their relative ease of isolation and robust viability for several days in culture; however, neonatal cardiomyocytes are more resistant to hypoxia than adult myocytes (236, 255), and the mechanisms of this resistance remain incompletely resolved, which may limit interpretation of such results when designing more translational studies (235). Adult myocytes are preferred, because ischemic heart disease almost exclusively occurs in adults, and fresh isolation eliminates the potential confounding factor of phenotypic transformation in culture. The caveat is that adult cardiomyocytes are more difficult to isolate and do not survive long in culture, impeding longer H/R protocols or those requiring pre-H/R transfection. Therefore, many investigators turn to neonatal cardiomyocytes or cell lines (e.g., H9c2 or HL-1) if genetic modifications are necessary, in which case these genetically engineered cells may be an important complement to in vivo or adult cardiomyocyte studies. In addition, adult myocytes do not form monolayers as seen with neonatal cells, limiting their use for studies on gap junctions and electrical conductivity. Thus, authors should carefully consider experimental end points in the context of overall study design, because either neonatal or adult cell sources will be the best choice depending on the question posed. For both neonatal and adult primary cells, isolation protocols must assure for a cardiomyocyte-enriched population (i.e., free of fibroblasts and other cell types). For neonatal cells, the differential attachment technique is often used, whereas gravity separation is typical for adult cardiomyocytes (161, 287). Regardless of the isolation and enrichment method, manual or automated cell counting, expression of cardiomyocyte-specific markers, and visualization or quantification of T-tubular structure should be performed to verify purity and phenotype. Use of iPSC-CMs may overcome some of these primary cell limitations, but the response of iPSC-CMs to H/R has not yet been fully characterized and may depend on their maturation state (256).

The most common in vitro conditions used to simulate in vivo ischemia are anoxia (<1% O_2_, 5% CO_2_, 94+% N_2_) and complete substrate depletion (serum-free, glucose-free medium). There are many variations to the protocol with additional conditions that more closely mimic the ischemic heart, such as partial hypoxia, partial or no substrate depletion, hyperkalemia, acidosis, and use of electrical stimulation (207). The cell type needs to be carefully considered when determining optimal control and ischemic conditions. Neonatal cardiomyocytes and cell lines (H9c2 and HL-1) favor glucose metabolism and under control conditions are often cultured in hyperglycemic medium, which is known to induce ROS production and cell death in adult cardiomyocytes. Furthermore, neonatal cardiomyocytes are insulin resistant and supraphysiological concentrations of insulin are required to increase glucose uptake in these cells compared with adult cardiomyocytes (201). Thus, studies focusing on metabolism with H/R and/or metabolic pathology need to carefully consider the cellular environment in both control and ischemic conditions. In addition to the cellular environment, the duration of hypoxia and reoxygenation is also an important consideration, as myocyte viability not only depends on the duration of hypoxia but also the duration of reoxygenation (166, 244).

To summarize, the major strengths and limitations of studies using isolated cardiomyocytes are shown in Table 1. The major strength of H/R experiments in cultured cardiomyocytes is the ability to control precisely the cellular and extracellular environment, each factor present in ischemic conditions (e.g., hypoxia, metabolic inhibition, or acidosis) can be tested alone and in combination to determine individual contributions to cellular injury. Even with the most carefully designed experimental protocol, in vitro conditions can never fully recapitulate the full spectrum of I/R injury in vivo. Thus, although in vitro experiments can be mechanistically informative and identify new targets for intervention, it is imperative that the results are later validated in an appropriate intact animal model. Nonetheless, even if there is a discrepancy between in vivo I/R and in vitro H/R experiments, important insight is to be gained from parallel studies. As an example, angiopoietin-like protein 4 (ANGPTL4) reduces infarct size after I/R in vivo but does not prevent cardiomyocyte death in vitro (90). These findings indicate that other cell types (e.g., endothelial cells, fibroblasts, or immune cells) are key to the cardioprotective effect of ANGPTL4. Likewise, factors that prevent myocyte cell death in culture may not reduce infarct size in vivo, suggesting that the cardioprotective effect may be outweighed by noncardiomyocyte factors.

### Isolated Perfused Hearts

#### Model rationale.

The isolated perfused heart is a convenient and reproducible model to test mechanisms of myocardial injury and cardioprotection (14, 192). The heart is removed from the animal and perfused, typically with a physiological saline solution such as Krebs-Henseleit buffer. For screening drugs or interventions for protective properties, this model is ideal, because the isolated perfused heart is studied independently of circulating factors or neuroendocrine inputs from other organs but retains the function, composition, and architecture of the intact heart. This approach is also easily amenable to biochemistry or imaging studies in a nuclear magnetic resonance (NMR) magnet, which can provide useful information to decipher mechanisms of cardioprotection.

Perfused hearts can be studied in a working heart mode or in a nonworking Langendorff mode. In the Langendorff mode, the perfusate enters the coronary arteries to perfuse and oxygenate the heart, which continues to beat for several hours (292). Heart rate and left ventricular (LV) developed pressure are measured with a fluid-filled balloon placed in the cavity of the LV and connected to a pressure transducer as indexes of cardiac function (physiology). The heart can be perfused at constant pressure, in which case the flow rate can vary, or flow can be set with a pump, in which case the perfusion pressure can vary. In the Langendorff mode, the heart does not pump against a gradient and does not perform external work (226). In the working heart mode, the perfusate enters the atrium at a filling pressure set by the operator, and the heart pumps perfusate against a hydrostatic pressure set to different levels (30). A heart model performing external work is technically more challenging, particularly with smaller hearts.

In a model of global ischemia, perfusate flow to the entire heart is stopped, whereas in a regional ischemic model, a suture is tied around a single coronary artery for occlusion. After the ischemic period (typically 20–40 min for rodent models), perfusion is restarted and the heart will usually beat and develop a lower LV developed pressure than at baseline, reflecting postischemic contractile dysfunction or stunning. Contractile dysfunction is a measure of ischemic injury but is not synonymous with cell death. Both contractile dysfunction and cell death often result from the same mechanisms. It is therefore important not to infer that protection against contractile dysfunction is the same as protection against infarct size.

#### Variables measured.

To measure cell death or infarct size, it is necessary to reperfuse the heart for a sufficient duration (at least 60–120 min) to wash out reductive equivalents (79, 271). Triphenyltetrazolium chloride (TTC) is then added to the perfusate or hearts are cut into transverse slices and incubated in TTC solution. TTC is a dye that stains viable myocardium red due to a formazan reaction with NADH and NADPH, which are washed out from irreversibly injured myocardium (81, 172), whereas necrotic tissue remains unstained and thus appears white. Necrotic tissue area is normalized to the total ventricular area (global ischemia) or the ischemic area at risk for infarction (regional ischemia). For regional ischemia preparations, the area at risk is measured after coronary reocclusion and staining of the nonischemic myocardium with a dye such as Evans blue.

The susceptibility of the heart to arrhythmias during I/R is readily assessed through recording of an electrocardiogram (313). Updated guidelines exist for the quantification of such arrhythmias (56). The isolated heart is amenable to monitoring of intracellular ions by optical methods using fluorescent indicators (where the signal originates from a thin layer of epicardial cells) or by NMR spectroscopy (where the signal is a global average from the whole heart). Intracellular Na^+^ and Ca^2+^ concentrations have been monitored and intracellular H^+^ concentration (i.e., intracellular pH) has been estimated in isolated rodent hearts perfused and subjected to I/R within the vertical bore of the NMR magnet (288). Intracellular high-energy phosphate (ATP and creatine phosphate) have also been monitored by this method, and recent developments using hyperpolarized substrates now also allow real-time analysis of metabolic flux through distinct pathways (167).

The rate of occurrence of cell death is determined by the work that the heart performs at the time it becomes ischemic. When global flow is stopped completely, the heart will continue to beat for a short period of time and continue to consume ATP. Reducing work at the start of ischemia is cardioprotective, and this is the basis of cardioplegic solutions. Therefore, it is important to assure that work is similar between control and experimental hearts, which means that heart rate and temperature must be controlled and held constant in the different treatment groups (281, 292). Due to the lack of neurohumoral factor influences on the perfused heart, heart rate is typically lower than in an intact animal, and it can be controlled by pacing. A slight (<1°C) difference in temperature can result in a large difference of infarct size. Temperature is usually measured by a probe in the heart and controlled by immersing the heart in a fluid bath.

In the absence of blood, the reduction in the oxygen-carrying ability of the saline perfusate results in edema and an increase in flow rate. Because the mouse has a high heart rate, it is likely that oxygen delivery is on the edge of oxygen demand under baseline perfusion conditions. Furthermore, Krebs-Henseleit buffer typically contains only glucose as a substrate, whereas the heart normally uses fatty acids as its prime substrate. Fatty acids can be given as substrates but require the addition of a vehicle such as BSA and also require specialized methods for oxygenating the buffer. An advantage of the perfused heart model is that it allows one to examine the impact of different substrates either alone or in combinations. The effect of differences associated with perfusion with long-chain versus short-chain fatty acids also can be studied. Ex vivo hearts can be readily perfused with radioactive- or stable isotope-labeled substrates, allowing evaluation of substrate selection and metabolism (167, 211, 245).

The no-reflow phenomenon (179) can also impact infarct size and its measurement in isolated hearts. During ischemia and early reflow, the heart goes into contracture, which restricts flow to the subendocardium. The severity of no reflow depends on the severity of ischemic injury and can vary between control and protected hearts. In isolated hearts, no-reflow issues can be reduced by deflating the balloon in the LV for a few minutes right at the start of reperfusion (94).

To summarize, the major strengths and limitations of the isolated perfused heart are shown in Table 1. In consideration for all of the above factors, it is imperative that control and treated hearts are studied under identical conditions. Although ischemic pre- and postconditioning were originally described in an in vivo dog model, much of the information about the molecular signaling pathways responsible for protection was established in perfused heart models (128).

## IN VIVO MODELS

### Chronic Coronary Artery Disease

#### Coronary stenosis and stress-induced myocardial ischemia: model rationale and variables measured.

Reversible episodes of ischemia can lead to contractile dysfunction in the absence of significant myocyte necrosis (42). Because ischemic entities are frequently encountered in clinical practice, the task of understanding their pathophysiology and testing therapeutic interventions has stimulated the development of animal models in which acute and chronic adaptations to ischemia and the ensuing functional recovery can be evaluated over time. Most of these entities are consequences of either brief or chronic episodes of ischemia, and the models developed to study them will be discussed separately below.

Chronic stable angina is a clinical condition whereby a patient has one or more coronary stenoses that have largely compromised or even exhausted autoregulatory coronary reserve. Frequently, these limitations are partially compensated by collateral blood flow from adjacent less-compromised coronary arteries such that myocardial blood flow and contractile function remain normal at rest. Stress situations such as exercise, emotions, or pain, however, can precipitate acute myocardial ischemia with or without typical chest pain. Chronic stable angina in patients does not usually inflict global myocardial ischemia but is a regional event. The acute precipitation of myocardial ischemia requires an in vivo model where an acute coronary stenosis can be produced to reduce coronary blood flow. Alternatively, a stable stenosis must be created where blood flow is maintained at baseline but acute ischemia is elicited, e.g., by pacing or adrenergic activation in anesthetized animals or by exercise in conscious animals (9, 93, 131).

To reflect the regional character of chronic stable angina, regional myocardial blood flow and regional contractile function must be measured. The standard approach to monitor regional blood flow is to use microspheres (142), which have traditionally been labeled with radioactive isotopes (66) and, more recently, nonradioactive colored dyes or fluorescent dyes (107, 184). Analysis of regional myocardial blood flow during acute ischemia reveals an inability of perfusion to increase distal to a stenosis compared with normal remote myocardium (33, 39). As coronary vasodilator reserve is exhausted, there is a major redistribution of blood flow away from the ischemic region toward the nonischemic myocardium where metabolic vasodilation prevails. In addition, there is a transmural blood flow redistribution from the ischemic subendocardium toward the subepicardium (93).

The gold standard for experimental regional contractile function measurements is sonomicrometry (265). Simultaneous measurements of regional myocardial blood flow and regional contractile function provide a means to determine the quantitative relationship between regional blood flow (as a surrogate for oxygen/energy supply) and regional contractile function (as a surrogate for oxygen/energy demand). The relation between regional contractile function and subendocardial perfusion (flow-function relation) demonstrates close coupling during steady-state ischemia at rest as well as over a wide range of cardiac workloads (33, 35, 37, 91, 309). During steady-state acute myocardial ischemia, there appears to be no imbalance between supply (blood flow) and demand (function); rather, there is a matched reduction in both parameters (129, 130). Such matching between regional blood flow and contractile function also persists during major changes in heart rate, when both blood flow and contractile function are normalized for a single cardiac cycle (92). This matching can persist for several hours and contribute to the maintenance of myocardial viability and full recovery of contractile function after eventual reperfusion (212). More specifically, the hallmarks of short-term myocardial hibernation are a perfusion-contraction match (259), together with metabolic signs of adaptation to ischemia (210, 267) and the potential to recruit an inotropic reserve in the dysfunctional myocardium (267). Also, all pharmacological interventions to attenuate acute myocardial ischemia (e.g., by nitrates, β-blockers, Ca^2+^ antagonists, or their combinations) operate along a fixed flow-function relationship (213–215). The two major mechanisms that precipitate myocardial ischemia and must therefore be pharmacologically addressed are tachycardia (112, 113) and coronary vasoconstriction (134, 273).

Studies evaluating brief total coronary occlusions can be conducted in a variety of species. In contrast, to study coronary artery stenosis, the animal under study must be large enough that coronary artery instrumentation along with regional flow and function measurements is feasible (i.e., in dogs and pigs that can be instrumented with a hydraulic occluder on the coronary artery). Acutely anesthetized animals have provided insight into short-term coronary flow regulation over minutes to hours but cannot evaluate the effects of chronic coronary stenosis on long-term microvascular remodeling and collateral growth over days to weeks (285). Acute experiments have the advantage that sequential myocardial biopsies can be taken for the analysis of metabolic and molecular analyses (101, 174, 210, 283). Microdialysis probes can be implanted to evaluate interstitial mediators (209, 270), and the activity of the cardiac innervation can be measured (132).

A significant experimental challenge is maintaining a fixed degree of coronary artery narrowing throughout a study using a hydraulic occluder. This limitation can by circumvented by perfusing the coronary artery at constant pressure from a reservoir, controlling flow with an extracorporeal pump or perfusion of the region of interest with an extracorporeal pressure control system (38). A major limitation of studies in acutely anesthetized animals are the substantial confounding effects of anesthesia and neurohormonal activation on hemodynamics and flow, which alter coronary autoregulation and produce varying degrees of coronary vasodilation and vasoconstriction that modulate autoregulatory responses (33, 36, 39). The limitations of acute studies can be circumvented by studying conscious, chronically instrumented animals. While strict control of hydraulic occluders and coronary collaterals stimulated by repetitive ischemia and chronic stenosis at first seems a limitation, these factors can be capitalized upon by provoking coronary collateral development to the point where the artery can be totally occluded without reducing resting myocardial perfusion. This is typically accomplished in dogs using ameroid occluders, which are hygroscopic and gradually swell to produce a progressive stenosis resulting in a total occlusion within 3–4 wk.

Collateral blood vessel growth can also be stimulated in dogs by repetitive brief coronary occlusions using a hydraulic occluder (303, 320). Once collaterals are developed, variability in the hydraulic stenosis severity is no longer a problem, and intervention effects on stress-induced ischemia can be studied under multiple conditions. While pigs can also develop collateral-dependent myocardium after ameroid occluder placement (260), collaterals grow more slowly than in dogs, and pigs frequently develop a subendocardial infarction (230). The admixture of infarcted and normal myocardium greatly complicates measurements of perfusion and function. Infarction can largely be circumvented by employing a fixed diameter stenosis on the coronary artery of farm-bred swine, resulting in a much more severe limitation of subendocardial flow reserve than in dogs, and there is usually contractile dysfunction at rest (74, 77). While this is an extremely useful model to study chronic vascular adaptations and interventions to promote angiogenesis, alterations in myocardial physiology can complicate the interpretation of flow changes. A major drawback of chronic large animal models of coronary stenosis and collateral-dependent myocardium is their expense and the labor-intensive nature of the animal care and handling. The guinea pig has a very well-developed collateral circulation that prevents infarction from occurring following occlusion of a single main coronary artery; to obtain infarction, multiple coronary arteries need to be ligated (216). Thus, guinea pigs are not suitable for in vivo ligation studies but can be used for heart perfusion with global ischemia experiments. These issues have motivated studies to assess flow regulation using repetitive coronary occlusions in rats and mice, including genetically altered animals (303).

#### Coronary microembolization: model rationale and variables measured.

Subclinical atherosclerotic plaque rupture or erosion that does not result in complete thrombotic occlusion of the coronary artery but leaves a residual blood flow into the distal coronary microcirculation occurs spontaneously, with or without clinical symptoms. Coronary microembolization is also induced iatrogenically during percutaneous coronary interventions. Atherosclerotic debris from the culprit lesion, together with thrombotic material, soluble vasoconstrictors, as well as thrombogenic and inflammatory substances, is washed into the coronary microcirculation where it causes microvascular obstruction with resulting patchy microinfarcts and an inflammatory reaction (135, 173). The inflammatory response includes increased expression of tumor necrosis factor-α associated with profound contractile dysfunction and upregulation of signal transduction pathways involving nitric oxide, sphingosine, and ROS, which contribute to impaired excitation-contraction coupling (32, 299). Repetitive coronary microembolization can result in global LV dysfunction and, even in the absence of overt infarction, in heart failure (261). Coronary microembolization can be simulated experimentally by intracoronary infusion of inert particles of various diameter (67) and also by intracoronary infusion of autologous microthrombi (191). When the target under study is ischemic heart failure, repeated coronary microembolization can be used in both small and large animal models. When the target under study is a spontaneous or periprocedural minor infarction, the animal must be large enough such that regional myocardial measurements of flow, contractile function, metabolism, and morphology are possible (i.e., dog or pig models are preferable).

The major strengths and limitations of angina models are shown in Table 1.

### Stunning, Hibernation, and Ischemic Cardiomyopathy

#### Stunning: model rationale and variables measured.

When ischemia caused by a total coronary occlusion is brief (e.g., as may be experienced from coronary vasospasm), regional contractile dysfunction persists for hours after reperfusion but then completely normalizes within 24 h. This phenomenon was first demonstrated after a 15-min circumflex coronary artery occlusion in chronically instrumented dogs, was subsequently called stunned myocardium, and is common in patients with acute coronary syndrome (17, 19, 143). Most investigators assume that the complete normalization of function, lack of evidence of infarction by TTC staining, and lack of sarcolemmal disruption on electron microscopy indicate that no cardiomyocyte death is associated with stunning. While pathological evidence of myocyte necrosis is indeed absent, TUNEL staining performed 1 h after reperfusion demonstrates that programmed cell death or myocyte apoptosis develops in rare isolated cardiac myocytes and circulating cardiac troponin I is increased (314). Thus, while there is no evidence of infarction in stunned myocardium, regional myocyte loss can develop when stunning becomes repetitive.

Because the essence of stunned myocardium consists of relatively rapid (24–48 h) reversibility of contractile dysfunction in the absence of TTC or pathological evidence of infarction, most studies use chronic large animal models in which serial measurements of function can be performed. In addition, regional ischemia is the preferred model to allow assessment of the remote nonischemic regions of the heart as an internal control. While stunned myocardium occurs after demand-induced ischemia distal to a coronary stenosis (144), most studies have used transient total coronary occlusion. Animals are instrumented with a hydraulic occluder to produce brief ischemia 1–2 wk after recovery from surgical instrumentation.

To assess regional function, most studies have used sonomicrometry for direct measurements of subendocardial segment length shortening or wall thickening. Recent studies have used transient occlusion of the left anterior descending coronary artery (LAD) using a balloon angioplasty catheter in closed-chest sedated animals where regional function can be assessed with imaging approaches such as echocardiography (314). The latter approach circumvents the need for chronic surgical instrumentation through a prior thoracotomy. Echocardiography can also be employed to assess stunning in mice chronically instrumented with an occluder to produce transient ischemia (63).

The dog (20, 143), pig (291, 314), and rabbit (18) are the most commonly used species to study myocardial hibernation. Pigs and rabbits offer the advantage of having little or no collateral circulation, so that the severity of the ischemic insult and of the subsequent contractile dysfunction are more uniform. In contrast, dogs exhibit a highly variable degree of collateral circulation resulting in widely different degrees of myocardial stunning (20). There are also species differences in the time course of recovery despite similar occlusion durations (277). Many studies have also used open-chest animal models, although the severity of myocardial stunning in these preparations is significantly exacerbated versus conscious animal models (21, 304). Most experimental approaches to assess stunning are quite straightforward, although ventricular fibrillation can develop. This is more common in swine as opposed to canine models, because pigs have little innate coronary collateral flow (164). Because myocardial function assessed using segment shortening and wall thickening is load dependent, it is important to ensure that heart rate, systolic blood pressure, and LV end-diastolic pressure remain reasonably constant over the time frame of the measurements. An advantage of chronic models using regional ischemia is that each animal can potentially serve as its own control; hence, it is possible to use the same animals to study the effects of pharmacological interventions on physiological end points.

#### Short-term hibernation: model rationale and variables measured.

A prolonged episode of moderately severe ischemia can be sustained for a period of hours in the absence of pathological evidence of infarction. This phenomenon is termed short-term hibernation (139, 212). An approximate 50% reduction in perfusion leads to reduced function and perfusion-contraction matching, which largely prevents irreversible myocyte injury. If the heart is reperfused within a few hours, contractile dysfunction persists in a fashion similar to stunned myocardium but with a more protracted time course of recovery (i.e., lasting in the timeframe of days rather than hours) as is typically seen with stunning after a brief total occlusion. This in part appears to relate to reversible myofibrillar disassembly and myolysis in the absence of sarcolemmal disruption (279). Unfortunately, when the initial adaptive response to moderate ischemia in short-term hibernation is present for longer than 12 h, progressive myocardial necrosis begins to develop, resulting in some degree of myocardial infarction usually confined to the subendocardium (48, 185, 269). While imposition of acute moderate ischemia was initially proposed as a mechanism of chronic hibernating myocardium, the development of progressive infarction when flow reductions last longer than 12 h leads to a pathological entity with myofibrillar disassembly and cardiac biomarker release that can no longer be defined as hibernation but, rather, is more in line with subendocardial infarction (48, 279).

Studies of short-term hibernation usually require closed-chest animal models, although considerable insight about adjustments between flow and function has been gleaned from open-chest studies of myocardial metabolism (137, 210, 239). The latter studies usually use a cannulated branch of the left coronary artery perfused at constant pressure. Closed-chest animal studies usually employ chronically instrumented dogs or pigs. In these studies, a hydraulic occluder is placed around a coronary to reduce flow or coronary pressure to a fixed level, which is released after several hours.

#### Chronic hibernation and stunning: model rationale and variables measured.

While both stunning and short-term hibernation are characterized by complete functional recovery, chronic contractile dysfunction can develop when recurrent ischemia develops before functional normalization (21). Chronic contractile dysfunction from repetitive ischemia develops in the absence of histological infarction, and both hibernation and stunning involve the loss of myocytes via apoptosis in a fashion similar to what happens after brief episodes of ischemia (193, 314). Unlike stunning, which was initially an experimental observation that later became associated with multiple clinical correlates, chronic hibernating myocardium was first characterized in patients with chronic ischemic heart disease displaying regional contractile dysfunction in the absence of manifest ischemia (23, 139). Only later were animal models used to identify cellular and molecular mechanisms responsible for the adaptive responses to chronic ischemia (77).

While it was originally controversial whether or not flow was reduced or normal at rest (34), it is now clear that chronic repetitive ischemia initially results in contractile dysfunction with normal resting flow or chronic stunning (73, 278). When this situation persists, the reduction in function leads to a secondary reduction in regional energy utilization accompanied by reduction in resting flow (76). Thus, the reduction in resting flow characteristic of chronic hibernating myocardium is a result, rather than cause, of regional dysfunction.

In contrast to models of short-term ischemia, animal models of hibernating myocardium are based on chronic coronary stenoses that frequently progress to total occlusion and collateral-dependent myocardium. In studies using ameroid occluders that gradually swell to produce chronic stenosis, dogs usually do not develop contractile dysfunction at rest but can do so when preexisting epicardial collaterals are ligated at the time of instrumentation (36). Swine ameroid occluder models frequently have contractile dysfunction in collateral-dependent myocardium, and this is usually associated with some degree of subendocardial infarction (230).

A more consistent model of hibernating myocardium can be produced by instrumenting juvenile swine with a fixed diameter stenosis (1.5-mm diameter) on the proximal LAD (73, 77). As the animals grow over the subsequent 3 mo, there is a slowly progressive limitation in coronary flow reserve, because the LAD stenosis limits maximal myocardial perfusion, while the mass of myocardium distal to the stenosis increases in parallel with cardiac growth. As a result, there is a more prolonged and gradual stimulus for coronary collateral development so that LAD occlusion almost always develops in the absence of infarction.

After 3 mo, regional contractile dysfunction with mild reductions of resting flow in the absence of infarction is consistently manifest and is similar to the changes seen in humans with hibernating myocardium caused by a chronic LAD occlusion (308). Serial studies of this animal model have demonstrated that the heart progressively adapts from a state of contractile dysfunction with normal resting flow (chronic stunning) to a state where resting flow decreases, consistent with hibernating myocardium (41). Such chronic hibernation is associated with a downregulation in mitochondrial metabolism and regional myocyte hypertrophy that maintains myocardial wall thickness constant in the setting of regional apoptosis-induced myocyte loss.

Over longer periods of time (up to 6 mo) the adaptive response of hibernating myocardium persists unchanged (75), and the downregulation in metabolism and upregulation of proteins involved in cellular survival and cytoprotection prevent cell death and, hence, further myocyte loss (62). While infarction does not develop in this model, revascularization only partially reverses myocardial dysfunction and does so over a much longer time frame than seen with either myocardial stunning or short-term hibernation (237). Chronic contractile dysfunction in the absence of infarction can also be induced using a hydraulic occluder to produce an acute stenosis in chronically instrumented animals. Chronic stunning can develop in swine subjected to daily episodes of short-term hibernation (169). A more rapid transition from chronic stunning to hibernating myocardium than the one observed in the fixed diameter stenosis model can be achieved by acutely imposing a critical stenosis on the LAD (301). The latter model can develop reductions in flow with regional contractile dysfunction after 2 wk of a stenosis sufficient to prevent reactive hyperemia.

The fixed diameter chronic stenosis model is advantageous in that hibernating myocardium develops reproducibly in a predictable time frame. A limitation of the fixed stenosis porcine model is that it requires cardiac growth to produce a progressive physiological impairment in maximum myocardial perfusion, and the 3- or 4-mo period required to develop hibernating myocardium is viewed as cost prohibitive. This model has so far only been studied in juvenile farm bred swine and may produce variable results if cardiac growth is attenuated by limiting feeding. It is not clear whether the model can be effected in purpose-bred swine and, particularly, in mini-swine, where growth rates are substantially attenuated. An additional disadvantage is that the chronic stenosis model is associated with a high rate of spontaneous ventricular fibrillation (40). This has provided insight into the mechanisms of sudden cardiac arrest in chronic coronary disease but reduced the success of studying chronic adaptations to ischemia in survivors. Finally, because of the long duration of the studies in the presence of animal growth, it is not feasible to chronically instrument animals. Nevertheless, it is feasible to use telemetry to assess chronically LV pressure and arrhythmias in untethered conscious animals (242).

#### Ischemic cardiomyopathy: model rationale and variables measured.

Ischemic cardiomyopathy is the underlying cause of LV dysfunction in two out of every three patients with heart failure (105). Ischemic cardiomyopathy in humans can arise from LV remodeling after a large myocardial infarction but, more commonly, is the result of extensive multivessel coronary artery disease with modest amounts of diffuse fibrosis and patchy infarction in multiple coronary artery distributions (15). Along these lines, preclinical studies have established that chronic coronary artery stenosis can induce significant myocyte loss with modest global replacement fibrosis that leads to global LV dysfunction and varying degrees of congestive heart failure when the area at risk is large. Conceptually, the stenosis does not limit blood flow at rest. Rather, by reducing maximal perfusion in response to stress, it sets the stage for repetitive episodes of subendocardial ischemia. A key feature of all animal models of ischemic cardiomyopathy is that the myocardium at risk of repetitive ischemia represents a large portion of the LV (>70% of LV mass). This has been achieved using stenosis of the left main coronary artery in rodents or multivessel coronary artery stenoses in large animals. As a result of the large area at risk, myocyte cell death arises from both ischemia and myocyte stretch and slippage from increased LV end-diastolic pressure (possibly also reflecting transient ischemia).

In rats, ischemic cardiomyopathy can be induced by producing a fixed coronary stenosis of ~50−60% diameter reduction on the proximal left coronary artery, which causes variable degrees of LV dysfunction (44, 45). While replacement fibrosis occurs in these animals, it is patchy and modest, only increasing twofold over control for an average of <10% of LV cross-sectional area. Interestingly, the degree of LV dysfunction is primarily related to myocyte cell loss (necrosis and apoptosis) and the elevation in LV end-diastolic pressure related to fibrosis. A similar model of ischemic cardiomyopathy has also been obtained in mice (189). While rodent models afford the ability to perform higher throughput studies and use transgenic animals to study molecular mechanisms, they have relatively high surgical and postoperative mortality. In addition, there is considerable variability in physiological outcomes, such that frequently animals are retrospectively categorized into mild, moderate, and severe heart failure groups. Reproducibility of ischemic cardiomyopathy models, therefore, is indeed a concern.

While left main coronary stenosis is not feasible in large animals, multivessel coronary stenoses can produce a large ischemic risk area and recapitulate ischemic cardiomyopathy. When fixed diameter occluders are placed on both the proximal LAD and circumflex arteries in growing farm-bred swine, LV ejection fraction declines with elevated resting LV end-diastolic pressure (74), consistent with compensated LV dysfunction and no overt evidence of heart failure. These animals also exhibit primary myocyte loss with only an approximately twofold increase in extracellular matrix accumulation. A similar condition has been produced using multivessel ameroid occluders in dogs (80). Aside from requiring survival surgery, the major disadvantage of these approaches arises from the development of sudden cardiac arrest, which in swine is related to both ventricular fibrillation and to a lesser extent bradyarrhythmias. In mice, a state of ischemic cardiomyopathy can be induced using repetitive brief coronary occlusions, and this model is associated with substantial but reversible fibrosis of the myocardial region subjected to repetitive ischemia (63).

#### Noninvasive imaging.

Noninvasive cardiac imaging technologies such as echocardiography, magnetic resonance imaging (MRI), and computed tomography can measure regional and global contractile function and are increasingly available for preclinical studies, particularly in larger animals. NMR spectroscopy can provide information on cardiac energetics (114). More sophisticated imaging technologies such as positron emission tomography can measure regional myocardial perfusion and regional myocardial metabolism and sympathetic innervation and are increasingly used in preclinical studies (77, 187, 268). MRI can serially measure myocardial perfusion (264) and can provide reliable measurements of infarct size and microvascular obstruction. MRI-derived edema, however, is time dependent and sensitive to cardioprotective interventions (141, 148). Therefore, MRI-derived edema can be used to stratify an ischemic/reperfused myocardial region for protocol assignment but not for quantitative normalization of infarct size to area at risk.

To summarize, the major strengths and limitations of stunning, hibernation, and ischemic cardiomyopathy models are shown in Table 1.

### Myocardial Infarction Models: Permanent Coronary Artery Occlusion with Nonreperfused and Reperfused Myocardial Infarction

#### MI: general considerations.

Coronary occlusion causes immediate cessation of aerobic metabolism in the ischemic myocardium, leading to rapid ATP depletion and metabolite accumulation and resulting in severe systolic dysfunction within seconds (86). If the duration of the ischemic insult is <15 min in larger mammals such as dog and pig, restoration of flow reverses the early ischemic cardiomyocyte changes (transient mitochondrial swelling or glycogen depletion) and all cardiomyocytes in the ischemic area can survive (158). Longer periods of ischemia cause death of an increasing number of cardiomyocytes. A 20- to 30-min interval of severe ischemia is sufficient to induce irreversible changes in some cardiomyocytes of the subendocardial area, inducing sarcolemmal disruption and striking perturbations in mitochondrial architecture, such as ultrastructural evidence of amorphous matrix densities and severe mitochondrial swelling (156). These early ultrastructural alterations mark cardiomyocytes that cannot be salvaged and will ultimately die in the infarct environment (157).

Experimental studies in the canine model of MI demonstrate a transmural heterogeneity in the myocardial response to ischemia, suggesting that the subendocardium, where myocardial oxygen demand is greatest, is more susceptible to ischemic injury than the midmyocardium and subepicardium (2). Thus, the prevailing paradigm suggests a wavefront of cardiomyocyte death that progresses from the more susceptible subendocardium to the less vulnerable subepicardium as the duration of the ischemic insult increases (159, 252). Experimental studies in large animal models have demonstrated that ischemic myocardium cannot be salvaged by reperfusion after 6 h of coronary occlusion (251). The increased vulnerability of subendocardial regions to coronary occlusion may reflect a greater reduction of the subendocardial blood flow due to transmural differences in vascularization (2, 25) and extravascular compression (68, 286). The wavefront concept of ischemia developing into infarction was derived from experimental studies in dogs, where a substantial coronary collateral circulation influences the time course of cardiomyocyte necrosis (86).

The major species difference in the MI response lies in the temporal and spatial kinetics of events and differences due to myocardial size. In mice, durations of coronary occlusion exceeding 60–90 min are considered irreversible, and inflammation and wound healing processes are accelerated (64, 88, 221, 222). In mouse and rat models, reperfused infarcts are typically midmyocardial, and subepicardial and subendocardial regions are relatively spared (50, 69, 325). Studies in a sheep model of reperfused infarction also suggest that the midmyocardium may be most vulnerable to ischemic injury; in contrast, the subendocardium is relatively resistant (263). The pig model of coronary occlusion-reperfusion comes closest to human STEMI in its temporal and spatial development, but other models are nevertheless useful to study fundamental mechanisms of MI (140).

#### MI: technical considerations.

Extensive protocols providing technical details for performing permanent occlusion MI and reperfused MI in mice and rats are available (221, 222, 228, 317, 327). While MI is most commonly performed in rodent models, protocols in other animal models are also available (151, 183, 218, 331). For mice, the quality of open-chest surgery to induce coronary occlusion directly impacts study outcomes (152, 221, 222). Minimizing the size of the thoracotomy and limiting bleeding by entering the thorax through intercostal muscles are recommended.

Biomarkers that have been used to evaluate the presence of MI include cardiac troponins and creatine kinase, and plasma proteins such as macrophage migration inhibitory factor can also be measured as indices of injury (47, 55). Infarct size is widely measured as a key variable for testing genetic or therapeutic intervention efficacy, and infarct size measurements taken serially at both early and late time points can evaluate the extent of infarct expansion (22). Echocardiography can also be used for infarct sizing, with the caveat that echocardiography does not distinguish between reversible ischemic dysfunction (stunning) and irreversible loss of function and therefore a secondary method is needed for confirmation of infarct size at early time points. For more details on measuring cardiac function in mice, the reader is advised to consult the article *Guidelines for measuring cardiac physiology in mice* (196).

#### Permanent occlusion MI: model rationale and variables measured.

Permanent coronary occlusion is a relevant animal model of acute STEMI reflective of patients who, due to contraindications or logistic issues, do not receive timely or successful reperfusion (53, 104). Permanent coronary occlusion yields acute ST segment elevation infarction with robust myocardial inflammation and long-term remodeling, thus providing a large effect size that reduces the sample size needed to detect differences between groups. Infarction assessed in the first 1–14 days after coronary ligation is histologically characterized by coagulation band necrosis with a fulminant inflammatory infiltrate in the infarct and border zone regions. Infarction is geometrically and physiologically characterized by wall thinning, increases in LV dimensions and volumes, and decreases in fractional shortening and ejection fraction.

Changes that occur over the first week provide information on myocyte cell death and infarct development, inflammation and leukocyte physiology, extracellular matrix (ECM) turnover and fibroblast activation, and the role of endothelial cells in neovascularization (83, 154, 165, 194, 205). Chronic evaluation at time points 4–8 wk post-MI provides information on long-term remodeling. Whether the infarct region or remote region is the focus of investigation depends on the question asked. Examining the infarct region provides details on active inflammation and scar formation, while examining the remote region provides details on still-viable myocytes within the myocardium and remote inflammatory and ECM processes.

Perioperative and postoperative survival should be assessed, and the time point of delineation between these two phases should be defined. For some laboratories, the perioperative phase includes the time until the animal recovers and becomes ambulatory (usually within 1–3 h for mice). For other laboratories, the perioperative phase includes the first 24 h after surgery. Perioperative death within 24 h post-MI in mice is usually due to surgical errors (or very large infarct sizes), and, in established laboratories, the 24 h surgical mortality rate due to technical issues is <10%. In the permanent occlusion MI model in mice, postoperative death (deaths at >24 h time point) typically occurs during *days 3–7* post-MI and is due to rupture, acute heart failure, or arrhythmias (59, 98, 233). Autopsy is strongly recommended for all mice that die prematurely, to evaluate early deaths due to technical issues and later deaths due to complications of MI. Seven-day postoperative mortality rates are ~10–25% (75–90% survival) for female young mice and 50–70% (30–50% survival) for male young mice (47, 61, 89, 98, 152, 170, 195, 202, 206, 234, 310–312, 319, 323). Immediate survival from the surgery can also be affected by baseline characteristics such as obesity, diabetes, or high levels of circulating inflammatory cells, which, in turn, determine the response to anesthesia and surgery (60, 123, 202, 203). While there is no difference in infarct tolerance between young and middle-aged mice (323), older mice may survive better than younger mice (202, 319).

For permanent occlusion MI models, infarct size must be measured in fresh LV slices at the time of necropsy by TTC staining and typically ranges from 30% to 60% of the total LV (47, 48, 61, 89, 98, 152, 195, 202, 206, 223, 310–312, 319, 323). The method for calculating infarct size varies across laboratories. Some laboratories use area calculations, other laboratories measure length in the midmyocardium, and other laboratories measure and average lengths in the subendocardium and subepicardium. There is no need to use Evans blue for area at risk assessment in permanent coronary occlusion models that pass the point from ischemia to infarction, as the entire area at risk is infarcted. It is important that MI surgical success is confirmed and that the initial infarct injury is comparable across groups, to assess remodeling differences at later stages. In mice, ligating the coronary artery at the same anatomical location across groups is important; 1 mm distal to the left atrium is the recommended site to generate large infarcts (35–60% of total LV). Failure to induce MI can occur, usually due to missing the coronary artery during the ligation step. Monitoring the electrocardiogram for ST segment elevation during the procedure reduces this possibility. Echocardiography at 3 h after coronary occlusion can be used to exclude animals with excessively small or large MI before randomizing groups (153, 155, 195). Late gadolinium-enhanced MRI is also useful for selecting animals with consistent infarct sizes (262). When assessing effects of treatments initiated post-MI, it is important to show that infarct size is not different between groups before treatment. Plasma sampling at 24 h post-MI can be used to assay cardiac biomarkers, such as troponins and inflammatory cytokines, with the caveat that these measurements can indicate presence or absence of infarct and not extent of injury. After coronary occlusion, care should be taken in performing these assessments to minimize disturbing animals at times when cardiac rupture may be triggered by stress, particularly at *days 3–7* post-MI in untreated controls (96, 98). Small infarcts may reflect technical issues in missing the coronary artery, resulting in damage from the suture rather than reflecting the intended myocardial ischemia and infarction. Infarct sizes <30% are typically excluded. If included, small and large infarcts may need to be grouped separately to reduce possible type II statistical errors.

Cardiac wound healing and remodeling, typically assessed days to weeks post-MI, can be examined using a wide variety of approaches, including echocardiography, histology, biochemistry, and cell biology (5, 217, 328). Serial measurements of cardiac geometry and function by echocardiography are useful for defining phenotypes. Cardiac dimensions vary depending on heart rate and depth of anesthesia, and these parameters must be carefully controlled and matched across groups. Cardiac functional reserve can be assessed by measuring the contractile response to inotropic drugs or volume overload. Cardiac MRI and hemodynamic assessment by pressure-volume catheterization are other ways to measure cardiac physiology parameters. It is feasible to quantify infarct size noninvasively and serially by using cardiac MRI (181). Hemodynamic evaluation in mice is a terminal procedure, which prevents its use in serial assessments.

Hematoxylin and eosin staining provides information on areas of necrosis and inflammation, while picrosirius red staining provides information on total collagen accumulation both in the scar and remote regions (316). Immunohistochemistry for neutrophils, macrophages, lymphocytes, fibroblasts, and endothelial cells provides information on the extent of inflammation, scar formation, and neovascularization. Isolating individual cell types and assessment of ex vivo phenotypes in culture can further aid in understanding mechanisms. Studies have revealed that inflammation evoked by acute myocardial infarction also occurs systemically and that the spleen and liver are important sources of cells and factors that influence LV remodeling (71, 72, 95, 116, 198, 199, 293).

#### I/R MI: model rationale and variables measured.

Implementation of myocardial reperfusion strategies has significantly reduced mortality in acute STEMI. Reperfusion has contributed to the growing pool of patients who survive the acute event and are at risk for adverse remodeling and subsequent development of heart failure (133, 136). In addition to salvaging cardiomyocytes, reperfusion has profound effects on cellular events responsible for repair and remodeling.

Although timely reperfusion is essential to salvage viable cardiomyocytes from ischemic death, extensive preclinical and clinical evidence suggests that reperfusion itself causes injury (119, 121, 147). Reperfusion-induced arrhythmias and myocardial stunning are self-limited and reversible forms of reperfusion injury, while microvascular obstruction and lethal cardiomyocyte injury are irreversible and extend damage, thus contributing to adverse outcomes following MI (13, 126, 177, 241, 318). In patients, no reflow during reperfusion may be exacerbated due to the generation of microemboli composed of atherosclerotic debris and thrombi during percutaneous coronary interventions (135, 253).

MI both with or without reperfusion shares many of the same technical guidelines, and this information is provided above. The one technical difference is whether the ligation is removed at 45–60 min after the occlusion to reperfuse the myocardium. Similar to permanent occlusion MI, studies investigating the inflammatory and reparative response following MI with reperfusion need to take into account the dynamic sequence of cellular events involved in repair. Common measurements shared by the two MI models are shown in Table 2. For studies aimed at investigating acute myocardial injury using a reperfusion strategy, the duration of coronary occlusion needs to be sufficient for the induction of significant MI but not overly prolonged to cause irreversible injury in the entire area at risk. From the cell physiology perspective, the reparative response after MI can be divided into three distinct but overlapping phases: inflammation, proliferation, and maturation (26, 65). In the infarcted myocardium, dying cardiomyocytes release damage-associated molecular patterns and induce cytokines and chemokines to recruit leukocytes into the infarcted region, thus triggering an intense inflammatory reaction that serves to clear the infarct from dead cells and ECM debris, while initiating a reparative response (84). Early reperfusion after irreversible cardiomyocyte injury accelerates and accentuates the inflammatory reaction and has profound effects on the pathological features of the infarct. Microvascular hyperpermeability is evident in the myocardium with acute I/R (97). Rapid extravasation of blood cells through the hyperpermeable vessels may result in hemorrhagic changes (98, 178). Influx of phagocytotic macrophages is accelerated, resulting in more rapid removal of dead cardiomyocytes compared with permanent occlusion MI. In reperfused infarcts, dying cardiomyocytes often exhibit large contraction bands, comprised of hypercontracted sarcomeres. Subsarcolemmal blebs and granular mitochondrial densities, which are already present in irreversibly injured cardiomyocytes before restoration of blood flow, become more prominent upon reperfusion.

**Table 2.** Common output measurements for in vivo MI and MI/reperfusion studies

Measurement	Information Provided
Infarct size	Infarct size (MI)
	Infarct size per area at risk (MI/reperfusion)
	Initial ischemic stimulus
	Final area of damage
	Effect of therapy or intervention
Plasma biomarkers	Ischemia: creatine kinase, troponins
	Inflammation: cytokines and chemokines
	Scar formation: growth factors and the ECM
	Neovascularization: angiogenic factors
Left ventricular physiology (echocardiography, MRI, positron emission tomography imaging)	Geometry and function: dimensions, wall thickness, left ventricular dimensions and volumes, fractional shortening, ejection fraction, remodeling index
	Electrophysiological function: PR, QRS, and QT intervals/morphology; spontaneous and inducible arrhythmias
Inflammation	Immunohistochemistry and immunoblot analysis for cell numbers and inflammatory protein expression
	Flow cytometry analysis of the digested myocardium for individual cell phenotypes
	Gene expression
	Systemic and circulating inflammation
ECM scar	Picrosirius red for collagen deposition
	Immunohistochemistry and immunoblot analysis for ECM proteins and cross-linking enzymes
	Gene expression
	Scar strength assessment
Neovascularization	Blood vessel numbers
	Vessel type and quality
Microvascular damage	Microvascular plugging
	Hyperpermeability/edema
	Hemorrhage

MI, myocardial infarction; ECM, extracellular matrix; MRI, magnetic resonance imaging.

Phagocytosis of dead cells by activated macrophages results in the activation of endogenous anti-inflammatory pathways, ultimately leading to resolution of the inflammatory infiltrate. Suppression of inflammation is followed by recruitment of activated myofibroblasts that deposit large amounts of ECM proteins and by activation of angiogenesis (145). As the scar matures, fibroblasts become quiescent and infarct neovessels acquire a coat of mural cells (332). Compared with large mammals, rodents exhibit an accelerated time course of infiltration with inflammatory and reparative cells (64).

Leukocyte infiltration during the inflammatory phase of infarct healing and myofibroblast activation and accumulation during the proliferative phase are predominantly localized in the infarct region and border zone (87, 122, 280). During scar maturation, the cellular content in the infarcted region is reduced. At the same time, however, the number of activated macrophages and fibroblasts in the remote remodeling myocardium increases. Therefore, study of inflammatory and reparative cell infiltration and assessment of ECM protein deposition should include systematic assessment of each end point in the infarcted region, the peri-infarct area, and the remote remodeling myocardium.

Sympathetic nerves are damaged by permanent coronary occlusion but can regenerate after injury (220). In the setting of chronic MI, regional hyperinnervation around the infarcted region has been observed, and activation of cardiac sympathetic nerves is important in triggering ventricular arrhythmias, and such proarrhythmic action is dependent on the extent of infarction (1, 70, 315, 326). In contrast, after I/R, chondroitin sulfate proteoglycans prevent reinnervation (99, 100). Thus, the model selected for sympathetic nerve evaluation should be taken into consideration and depends on what question is being asked.

#### MI: intervention considerations.

The effects of interventions on post-MI remodeling can be studied using both nonreperfused MI and reperfused MI/R models (11, 219, 232, 294, 322). Typically, nonreperfused MI yields accentuated dilative remodeling and exacerbated dysfunction compared with a reperfused infarct involving the same vascular territory, reflecting a combination of more extensive infarct and less effective repair. In the reperfused MI/R model, the effects of genetic or pharmacologic interventions implemented early after reperfusion may reflect differences in the extent of acute cardiomyocyte injury rather than differences in wound healing responses. With permanent occlusion MI (assuming a standardized area at risk) or very late reperfusion models, differences in geometry and function of the remodeling heart are independent of acute cardiomyocyte injury and reflect effects on inflammatory, reparative, or fibrotic cascades. In the presence of an occluded coronary artery, the delivery of systemically administered pharmacologic agents to the infarcted region of large animal models may be dependent on formation of collaterals.

While the development of genetically targeted animals (mice, rats, and rabbits) resulted in an explosion of studies dissecting cell biological mechanisms and molecular pathways, large animal models are considered closer to the clinical situation for translational studies to test safety and effectiveness. Optimal study of molecular, cellular, and LV functional end points and interpretation of the findings require understanding of the underlying pathophysiology. Assessment of infarct size is typically the primary end point for investigations examining the mechanisms of cardioprotection. Assessment of chamber dimensions using echocardiography or MRI is crucial to study the progression of adverse remodeling. Systolic and diastolic cardiac geometry and function can be assessed noninvasively using echocardiography (including Doppler ultrasound and speckle tracking), MRI, and hemodynamic assessment. Mechanistic dissection of specific pathways may require inclusion of additional cell physiology and molecular or proteomic end points. In experimental models of MI, understanding the time course of the cellular and molecular events is critical for optimal study design. The effects of varying ischemic intervals on survival and activation of noncardiomyocyte cellular and acellular (e.g., ECM) compartments are poorly understood. Longer coronary occlusion times have distinct effects on cardiac repair, by extending infarct size and by influencing susceptible noncardiomyocyte populations, such as endothelial cells, fibroblasts, pericytes, and immune cells (85). Most studies characterizing responses to myocardial injury have so far been performed in healthy young animals. Comorbid conditions, such as aging, diabetes, and metabolic dysfunction, affect the pattern of ischemic injury and modify the time course and qualitative characteristics of the inflammatory and reparative responses (27, 106, 202, 238, 298, 319, 323). These comorbidities are relevant in the clinical context and must be considered in translation of experimental findings to the clinic.

To summarize, the major strengths and limitations of the nonreperfused and reperfused MI models are shown in Table 1.

### Ablation

#### Model rationale and variables measured.

The primary advantages of ablative injury techniques such as cryo-, thermal-, and radio-frequency ablation are rigid and reproducible control over the size, shape, and location of the region of damage. With such methods, a wound can be stamped on the target myocardial tissue with consistent dimensions, shape, and transmural depth. Because the size of the damaged region is independent of animal-to-animal variations in coronary anatomy (223), the resultant ablation scar is also more reproducible than ligation-induced injury (52, 160, 307), aiding studies of long-term structural and functional remodeling and providing better power to detect the effects of an experimental drug or cell therapy. Infarct location can thus be controlled independently from coronary anatomy and infarct transmurality can be controlled (52, 160, 290, 305, 307). There are, however, important differences in the modes of cell death in ablative vs. occlusion injuries. For example, cryoinjury results in necrosis due to the generation of ice crystals and disruption of the cell membrane rather than direct ischemia. Furthermore, ablative injuries are typically generated from the epicardial surface inward, whereas ischemic infarcts tend to be propagated outward from the inner myocardial layers (52, 160).

Unlike MI or MI with reperfusion, cryoinjury kills all (or nearly all) cells within the core of the damaged region and creates distinct wound margins. Thus, a number of studies have used cryoinjury to avoid confounding effects of resident surviving cells when testing stem cell and other related therapies (6, 7, 258, 302). Ablation procedures typically apply a cooled/heated probe to either the epicardial or endocardial surface of the heart. The extent and depth of the lesion depend on both the temperature of the probe and the time it remains in contact with the tissue; damage can be extended by generating multiple adjacent lesions or by repeat application at the same location. Because these physical factors are central to injury formation, investigators should report probe size and material, temperature, method and duration of preheating/cooling, precise anatomic location and duration of probe application, and interlesion time and number of lesions (if applicable). Cryoablation has been used to generate reproducible wounds and scar tissue for the study of myocardial injury response in various species including dogs (160, 171, 297), rabbits (6, 7, 302), rats (49, 82, 149, 190), and mice (109, 204, 257, 289, 305, 307). In mice, survival rate after cryoinjury was nearly twice that of permanent coronary ligation over an 8-wk period (307), whereas dysfunction was similar. Lower mortality may be a consequence of smaller infarct size. Ablative methodologies have also been used in nonmammalian species such as zebrafish, to probe the response of cardiac electrical properties to injury, regeneration and scar formation (43, 46, 108). The ability to destroy all cells within the cryoinfarct has provided interesting clues regarding regeneration of fetal myocardium following injury. In neonatal mice, mechanical or ischemic injuries to the ventricular apex typically trigger regeneration, producing recovery of myocardial structure and function without scarring (243, 276). Nontransmural cryoinfarcts in neonatal mice similarly heal with minimal evidence of scarring and full functional recovery with ongoing postnatal growth, while injuries spanning the full thickness of the ventricular wall do not regenerate muscle (57). Because neonatal mice can regenerate myocardium during the first postnatal week, models of myocardial ischemia in neonatal mice may be used to identify pathways involved in cardiac regeneration (8, 208).

As with coronary ligation models, evaluation of LV geometry and function with echocardiography and assessment of electrophysiological remodeling and arrhythmia risk are routinely performed in cryoinjury models. Given the early time course and different mechanisms of necrotic injury in cryoinjury versus ligation models, cryoinjury studies are typically more focused on long-term myocardial regeneration or mechanical/electrophysiological remodeling rather than mechanisms of acute postinjury cell death, inflammation, and scar formation (52, 160). Ablation procedures are now used commonly in clinical electrophysiology, and it is therefore not surprising that a number of experimental electrophysiology studies have taken advantage of geometric control provided by this model (54, 231). Cryoinjury and ischemic injury differ in their transmural localization and the amount of surviving myocardium (52, 109, 257, 258, 305). Finally, methods for ablative targeting of cardiac neural tissues have proven useful in studies of the role of autonomic inputs in normal and arrhythmic hearts (254).

To summarize, the major strengths and limitations of the cryoinjury model are shown in Table 1.

### Cardioprotection

#### Model rationale and variables measured.

An intervention that is cardioprotective is broadly defined as serving to protect the heart (https://www.merriam-webster.com/dictionary/cardioprotective), thereby in theory encompassing all of the aforementioned aspects of cardiac damage and dysfunction. To date, the only clinically established cardioprotective intervention is early reperfusion (119, 133). The archetypal additive cardioprotective intervention is, without question, ischemic conditioning, encompassing the phenomena of ischemic preconditioning, postconditioning, and remote conditioning (118, 175, 224, 247, 248, 329). Despite differences in the timing of the protective stimulus (with preconditioning applied in a prophylactic manner and postconditioning administered at the time of reperfusion) and the site of the protective trigger (either locally, or, for remote conditioning, in a tissue or organ distant from the at-risk myocardium), all three forms of conditioning share a common theme: there is overwhelming agreement that ischemic preconditioning, postconditioning, and remote conditioning render the heart resistant to lethal I/R-induced injury (78, 118, 128, 163, 247).

This consensus with regard to ischemic conditioning and cardioprotection is, however, a notable exception in the field. Indeed, for the vast majority of the innumerable cardioprotective strategies that have been investigated, the current preclinical literature on the topic of cardioprotection is fraught with controversies and a lack of reproducibility among investigators and laboratories (163, 188). The ensuing confusion in the field may be attributed in part to two confounding factors: an overly broad use of the term cardioprotection in some studies, together with false positive and false negative outcomes derived from protocols executed in a suboptimal manner (162, 163, 188).

In some instances, a broad and suitably framed definition of cardioprotection incorporating, for example, endothelial integrity and vascular function, is appropriate (126, 225). A generally acknowledged and more narrowly focused hallmark of cardioprotection is defined as an agent or intervention that, when administered in the setting of ischemia/reperfusion, augments myocardial salvage and reduces myocardial infarct size beyond that achieved by reperfusion alone (128). Accordingly, for our purposes, we focus on cardiomyocyte viability and define cardioprotection as a strategy that attenuates cardiomyocyte death. Cardiomyocyte viability can be assessed in a full spectrum of models, ranging from cardiomyocytes in culture to isolated buffer-perfused hearts to in vivo studies in rodents (mice and rats) or larger animals (including rabbits, dogs, pigs, sheep, and, in a small number of studies, primates). There is no ideal model that completely mirrors the clinical scenario to fully ensure absolute translational relevance. Rather, each model has merits and disadvantages (as shown in Table 3).

**Table 3.** Recommendations for cardioprotection studies

Model	Gold Standard Primary End Point	Required Covariates	Potential Secondary End points	Advantages	Limitations
Cultured cardiomyocytes	Cell viability (live-dead assay)	None	Types of cell death (i.e., apoptosis), mitochondrial function; ROS production	Capacity for high throughput	Reductionist
Isolated buffer-perfused hearts (mouse, rat, and rabbit)	Infarct size (TTC)	For models of regional ischemia: area at risk	Measures of LV function and coronary flow; cTn as a secondary index of cardiac injury	Throughput higher than in vivo; eliminates confounding effect of intervention on systemic blood vessels	Reductionist
Mouse	Infarct size (TTC)	Area at risk; hemodynamics	Measures of LV function; measures of no reflow; CK or cTn as secondary indexes of cardiac injury	Availability of genetically modified strains; model of low collateral flow (measurement of RMBF not required)	Small size; differences between strains; substantial variability requiring large *n* values
Rat and rabbit	Infarct size (TTC)	Area at risk; hemodynamics	Measures of LV function; measures of no reflow; CK or cTn as secondary index of cardiac injury	Reliable infarct production; relatively high survival rate in experienced hands; commercial availability of strains that have comorbidities; model of low collateral flow (measurement of RMBF not required)	Not high throughput
Dog	Infarct size (TTC)	Collateral blood flow; area at risk; hemodynamics	Measures of LV function; measures of no reflow; CK or cTn as secondary indexes of cardiac injury	Large amount of historical data; shows effect of intervention in the setting of variable collateral flow; mimics humans with high collateral flow	High cost; variability in collateral perfusion (RMBF must be measured); potential for lethal arrhythmias; not high throughput
Pig	Infarct size (TTC)	Area at risk; hemodynamics	Measures of LV function; measures of no reflow; CK or cTn as secondary indexes of cardiac injury	Model of low collateral flow (measurement of RMBF not required); mimics humans with low collateral flow	High cost; high incidence of lethal arrhythmias; not high throughput

TTC, triphenyltetrazolium chloride; LV, left ventricular; CK, creatine kinase; cTn, cardiac troponin; RMBF, regional myocardial blood flow.

The overwhelming strength of the mouse species is the availability of genetically modified strains to elucidate molecular mechanisms once candidate cardioprotective strategies have been identified, a benefit that is balanced by inherent variability and resultant requirement for large sample sizes. An additional problem of the mouse is the atypical geometry of nontransmural infarcts, in which the subendocardium is spared from death by diffusion of oxygen from the LV cavity and occupies an inordinate proportion of the total LV wall thickness. In all rodents, heart rate is much higher and therefore infarct development is much faster than in larger mammals and humans. Studies conducted in large animals (in particular, the pig) are considered to have the greatest potential for preclinical relevance (103, 140). This advantage is accompanied by substantial costs incurred and, particularly in pigs, the well-documented high incidence of lethal ventricular arrhythmias (282).

In all studies focused on cardioprotection, the primary end point must be a quantitative assessment of cardiomyocyte viability. In cell culture models, these data may be obtained using a live-dead assay such as trypan blue exclusion, propidium iodide exclusion, or other commercially available assays. In intact hearts, including isolated buffer-perfused hearts and all in vivo models, the gold standard end point is myocardial infarct size by TTC staining and, at later time points, histopathologic analysis (79, 81). For models involving reperfusion, the area at risk must be quantified and infarct size must be expressed as a proportion of the risk region. With global rather than regional ischemia/reperfusion, the entire heart is rendered at risk and thus infarct size is appropriately expressed as a proportion of the total ventricular area. The duration of ischemia must be sufficient to cause significant infarction but not complete death of at-risk cardiomyocytes in the control cohort. That is, if the duration of ischemia is selected such that infarct size in controls is either inordinately small or excessively large, the scope for salvage and probability of achieving cardioprotection with any intervention is negligible. Irrespective of the model used, the protocol must involve I/R (or, in cell culture models, H/R) rather than ischemia (or hypoxia) alone. This requirement reflects the fact that even the most powerful and well-established cardioprotective strategies such as ischemic preconditioning simply delay, rather than prevent, the progression to cardiomyocyte death and infarction (224, 324). The duration of reperfusion must be sufficient to allow for the accurate and unambiguous delineation of necrotic and viable myocardium. This is of particular importance when infarct size is quantified using TTC staining: a minimum of 1–2 h of reperfusion is considered mandatory in rodent hearts, while longer periods of at least 3 h are standard in large animal models (283).

Attention must be paid to essential covariates and possible cofounders. Important considerations for all in vivo models include body temperature, the choice of anesthetics and analgesics, and changes in the determinants of myocardial oxygen supply and demand, all of which are well recognized to have profound effects on infarct size (111, 247). Particular care must be taken to avoid the possibility of inadvertent preconditioning: examples include triggering a protective phenotype by unintentionally subjecting the myocardium to brief periods of hypoxia or ischemia during surgical preparation, or intentionally imposing a period of ischemia in an effort to identify the extent of the at-risk myocardium (180). Additional covariates and confounders are model specific. In rodents, age, sex, and strain of the animals have all been implicated or identified to influence myocardial infarct size (10, 111, 306) Circadian variation, the time of day at which experiments are performed, may also be important (16, 28, 110, 266). The canine model is known for its variability in the magnitude of collateral blood flow. Accordingly, when using this model, measurement of regional myocardial blood flow during coronary artery occlusion and incorporation of collateral flow as a covariate in the analysis of infarct size are mandatory (64, 284).

Finally, the overwhelming majority of studies conducted to date have assessed the efficacy of candidate cardioprotective strategies using healthy, juvenile, or adult animals. There is evidence that the infarct-sparing effect of these purportedly protective interventions may be lost or attenuated in the setting of clinically relevant comorbidities, including aging, type 1 and type 2 diabetes, hypercholesterolemia, and hypertension, and may be influenced by diet or exercise (3, 4, 51, 78, 115, 117, 146, 150, 186, 197, 246, 249, 321). Accordingly, once proof of principle is established, it is imperative that promising cardioprotective therapies be reevaluated, adhering to the essential elements of rigor described above, in comorbid models (127). All protocols must include concurrent and appropriate control cohorts. For example, when potential cardioprotective drugs are evaluated, controls must receive matched volumes of vehicle administered in an identical manner.

## THE ISSUE OF TRANSLATION: TOWARD A RANDOMIZED CONTROLLED STUDY OR TRIAL DESIGN

There is currently no established intervention, aside from timely reperfusion, that limits damage to hearts of patients experiencing myocardial ischemia to the extent that clinical outcome is improved (133, 138). Discussions of prior failures and hope for future successes have been reviewed elsewhere (120, 127, 176). Several elements missing from preclinical studies of infarct size reduction have been identified and include absence of critical investigator blinding, statistical weaknesses (underpowered studies), and insufficient methodological detail. These deficiencies explain in part the failure to translate preclinical results into effective infarct-sparing treatments in patients. Thus, many have questioned the reproducibility of interventions to protect from MI (i.e., reduce infarct size).

Much of the lack of reproducibility has been ascribed to limited or lacking scientific rigor (29, 250). In response to these and other concerns, the United States National Institutes of Health now includes explicit requirements for applicants to show, and reviewers to evaluate, the level of scientific rigor in grant applications. Whether suboptimal rigor fully explains and underlies the reproducibility crisis is unclear (162). Nonetheless, advocating for reproducibility and scientific rigor is welcome.

Appropriate statistical issues should be considered before initiating an infarct-sparing intervention. Investigators should know the standard deviation of their primary, prespecified end point, such as infarct size, chamber dimension, or ejection fraction. A power analysis for the primary end point will determine and justify the number of subjects to be enrolled in each group. This may not be feasible when investigating an entirely innovative strategy as there may be no basis for an estimation of the expected effect size. The standard deviation of the investigator’s most recent blinded study can be used to determine group size (229). The choice of statistical analyses must be appropriate for the study design (300). For two-group studies, *t*-tests (or nonparametric equivalent) may be used; for protocols involving multiple cohorts, ANOVA (or nonparametric equivalent) is mandatory. Analysis of covariance, assessing the effects including variations in risk region and collateral blood flow, may also be applied.

Blinded assignment of animals and randomization to control or treated groups is mandatory whenever possible. Incorporation of randomization with blinding is an easy and logical approach. For testing classical drug-based interventions or when comparing mutant mice, individuals responsible for blinding can use block randomization and label tubes or mice with a simple unique alphanumeric code. The surgeon performing the protocol should have no knowledge of the intervention or genotype. We recommend that the same surgeon perform all surgeries; if multiple surgeons are used, equal numbers from all groups should be matched across the surgeon pool. It is imperative that neither the individual analyzing infarct size, nor the individual performing any other secondary analyses, knows the intervention or genotype until all data are compiled. If the potential for excluding animals exists, this should be done based on previously declared exclusion and inclusion criteria, and all decisions should be made before disclosure of group assignment; details of such exclusions should be made clear in any publications. Table 4 shows ARRIVE guidelines for manuscript submission, modified to focus on ischemia studies.

**Table 4.** Checklist of considerations for rigor and reproducibility, modified from the ARRIVE Guidelines (168)

Item	Details
Ethical statements	Institutional Animal Care and Use Committee approval, *Guide for the Care and Use of Laboratory Animals*, welfare assessments and interventions
Animal description, housing, husbandry	Species, strain, source, age (mean and range), sex, genotypes, body weight (mean and range), health/immune status, housing type (specific pathogen free), cage type, bedding material, number of cage companions, light-dark cycle, room temperature and humidity, food type, food and water access
Study design	Define model used; define groups; matching, randomization, and blinding protocols; order of treatment and assessment of groups; method to confirm model success; inclusion/exclusion criteria (e.g., lower limit for infarct size); daily monitor to record time and cause of death; define timing of perioperative and postoperative phases for survival analysis, flow chart for complex designs
Experimental procedures	Define area examined (e.g., infarct, remote, both regions); positive and negative controls; for drugs: formulation, dose, site, and route of administration; anesthesia and analgesic use and pain monitoring; surgical procedure details and monitoring records (e.g., electrocardiogram, heart rate, and anesthesia depth); method of euthanasia; time of day performed
Sample sizes	Number of animals used per group for each experiment; sample size calculation; number of independent replicates for cell culture studies
Variables measured	Primary and secondary end points, list any animals or samples removed from analysis with reason
Statistics	Methods used for each analysis; test for assumptions

ARRIVE, Animals in Research: Reporting In Vivo Experiments.

The Consortium for Preclinical Assessment of Cardioprotective Therapies (CAESAR) endeavored to address the primary issues related to reproducibility in cardioprotection studies (163). Performing the same protocol at multiple sites and in multiple species was extraordinarily challenging; the most notable were challenges in identifying the underlying explanations for differences in infarct size (or other variables) between centers while they were implementing consonant protocols. Interestingly, there were several instances of mice being ordered simultaneously from the same vendor, only to have significantly different body weights at the time of study (despite being fed the same chow). To be clear, the weight differences were relatively small but on occasion were significantly different for reasons unknown. It is likely that small differences, such as this, could theoretically affect the results (or the perception of not being able to reproduce studies) of published studies. During the CAESAR experience, numerous seemingly extraneous factors were considered, such as differences in municipal water source, room temperature, relative humidity, type of lighting, traffic in the room, and other seemingly innocuous details that may or may not affect the responsiveness of mice to an infarct-sparing regimen. All of these variables reflect inherent challenges in performing in vivo studies.

More important than slight differences in body weight or other such ancillary factors were initial challenges in generating equivalent infarct sizes at different institutions, despite using the same protocol. Such occasional variations emphasize the unequivocal requirement in all protocols for concurrent and appropriate controls (see *Cardioprotection*).

## OVERALL DISCUSSION AND CONCLUSIONS

As highlighted throughout these guidelines, ischemia and I/R have multiple consequences that show temporal variation in terms of both incidence and influence on outcomes and may be model dependent. Examples range from acute effects (including biochemical perturbations in cardiomyocytes and other cardiac cell types, disruption in cardiac conduction and development of arrhythmias, contractile dysfunction, abnormalities in endothelial, and vascular reactivity) to longer term outcomes (such as cardiomyocyte death, microvascular obstruction and no-reflow, scar healing, and LV remodeling) and, ultimately, major adverse cardiac events, including heart failure and death (136). Figure 1 shows the diversity in models available to assess ischemia across its spectrum, and Tables 4 and 5 show general recommendations for rigor and reproducibility in ischemia studies.

**Table 5.** Recommendations for ischemia studies

Common	Experimental design should follow ARRIVE guidelines (see Table 4)
Cardiomyocytes	When comparing groups, geometry and function end points by echocardiography assessment may not change. This does not necessarily indicate no effect of the intervention.
	Control groups can be shared across studies to reduce animal use, as long as the samples are collected within the same timeframe, under identical conditions, and details are provided in the methods. Previously collected historical controls should be avoided or clearly indicated.
	For time-course studies, sham surgery can be replaced by *day 0* negative controls if minimally invasive procedures are used, which greatly reduces animal use.
	Use to discern direct cardiomyocyte effects and responses
Isolated perfused hearts	Use to discern cardiac effects and responses
Angina, stunning, hibernation, and ischemic cardiomyopathy	Use to reflect a particular clinical scenario
MI	Use nonreperfused or reperfused MI to study repair and remodeling
	Use nonreperfused MI to test interventions in a robust remodeling model and to test interventions in a model clinically relevant to the nonreperfused patient
	Use reperfused MI to test interventions in a model clinically relevant to the reperfused patient
	Use reperfused MI to study cardioprotection
	Essential to measure infarct size for nonreperfused MI and infarct size and area at risk for reperfused MI
Ablation	Use to control size, shape, or location
	Use to achieve maximal and uniform cell death in the target region
	Use to investigate mechanisms of action of corresponding to clinical ablation technique
	While not suited to study MI pathophysiology, is well suited to study repair and regeneration
	Essential to quantify transmural extent of damage, to assess transmural variation
	Essential to recognize that transmural extent of the lesion may evolve over time
	Essential to standardize experimental protocol (e.g., probe temperature and contact time) to achieve consistent lesions
Cardioprotection	Use to evaluate potentially protective strategies in the ischemia-reperfusion model
	Essential to measure infarct size and area at risk

ARRIVE, Animals in Research: Reporting In Vivo Experiments; MI, myocardial infarction.

While writing these guidelines, the authors discussed whether an algorithm to define the choice of model for a given scientific question would be helpful. The consensus was that the topic of myocardial ischemia and infarction and the many clinical manifestations of coronary artery disease resulting in and from myocardial ischemia or infarction are so broad and so complex that we find ourselves unable to provide an algorithm that truly covers all potential scientific approaches. Indeed, such an algorithm may be used by regulatory and funding agencies to actually limit research in the field, which would be counterproductive to the goals of this document.

The approach used will vary depending on the questions being addressed; as such, all of the approaches described above may be considered good and a gold standard if appropriate to address the target hypothesis. In vitro studies using isolated cardiomyocytes or even isolated organelles (e.g., mitochondria) are well suited to identify single molecular targets of injury and protection (102). Isolated, buffer-perfused heart models can be used to study the acute biochemical and functional mechanisms of myocardial I/R injury and cardioprotection. Due to the need for stable stenosis and the required spatial resolution of regional myocardial blood flow and contractile function measurements, large animal preparations are recommended as models for the clinical manifestations of chronic stable angina and coronary microembolization. In vivo preparations are required to study more long-term effects of myocardial I/R and respective therapeutic interventions. We distinguish between permanent occlusion MI and reperfused MI models and also highlight measurements in common. Permanent coronary occlusion MI and reperfused MI models are both well suited to study repair and remodeling. I/R models are mandatory to study cardioprotection, and the ischemia must be of sufficient severity and duration to cause some infarction. Cryo-/thermoinjury is not suited to study the pathophysiology of MI but well suited to study repair and regeneration processes.

Prospective planning of study design, i.e., randomization for appropriate control versus treatment, blinding of investigators (as much as possible for a given experimental protocol and the subsequent data analysis), and adequate statistics, is mandatory for reproducibility of all experimental models of myocardial I/R and infarction. As larger data sets are being acquired, consideration for how to harness big data should be given (272, 274). Compiling databases to incorporate results from across studies and across laboratories will provide a means to use epidemiological approaches or big data tools to validate published findings, generate novel hypotheses, and assess individual variability in response to ischemia.

In conclusion, these guidelines provide recommendations to help the investigator plan and execute a full range of studies involving myocardial ischemia and infarction.

## GRANTS

We acknowledge support from the following: National Institutes of Health Grants HL-002066, HL-051971, HL-056728, HL-061610, HL-075360, HL-078825, HL-088533, HL-092141, HL-093579, HL-107153, HL-111600, HL-112730, HL-112831, HL-113452, HL-113530, HL-116449, HL-128135, HL-129120, HL-129823, HL-130266, HL-131647, HL-132075, HL-135772, GM-103492, HL-131647, HL-76246, HL-85440, GM-104357, GM-114833, GM-115428, and UL1-TR-001412; American Heart Association Grants 16GRNT30960054 and 16CSA28880004; Grand Challenge Award; Department of Defense Grants 16W81XWH-16–1-0592, PR151051, PR151134, and PR151029; Biomedical Laboratory Research and Development Service of the Veterans Affairs Office of Research and Development Awards 1IO1BX002659 and I01BX000505; Australian National Health and Medical Research Council Research Fellowship APP1043026; Bundesministerium für Bildung und Forschung Grant BMBF01 EO1004; and German Research Foundation Grants DFG He 1320/18-3 and SFB 1116 B8. R. A. Kloner reports grant support from Stealth Biotherapeutics, Servier, Inc., and Faraday to test experimental compounds in experimental myocardial infarction models.

## DISCLAIMERS

The content is solely the responsibility of the authors and does not necessarily represent the official views of the National Institutes of Health, American Heart Association, United States Department of Defense, United States Veterans Administration, National Health and Medical Research Council, or German Research Foundation.

## DISCLOSURES

No conflicts of interest, financial or otherwise, are declared by the authors.

## AUTHOR CONTRIBUTIONS

M.L.L. and G.H. conceived and designed research; M.L.L., R.G.G., K.P., C.M.R., and G.H. prepared figures; M.L.L., R.B., J.M.C., X.-J.D., N.G.F., S.F., R.G.G., J.W.H., S.P.J., R.K., D.J.L., R.L., E.M., P.P., K.P., F.A.R., L.S.L., C.M.R., J.E.V.E., and G.H. drafted manuscript; M.L.L., R.B., J.M.C., X.-J.D., N.G.F., S.F., R.G.G., J.W.H., S.P.J., R.K., D.J.L., R.L., E.M., P.P., K.P., F.A.R., L.S.L., C.M.R., J.E.V.E., and G.H. edited and revised manuscript; M.L.L., R.B., J.M.C., X.-J.D., N.G.F., S.F., R.G.G., J.W.H., S.P.J., R.K., D.J.L., R.L., E.M., P.P., K.P., F.A.R., L.S.L., C.M.R., J.E.V.E., and G.H. approved final version of manuscript.
